# Virulence Profiles, Phylogenetic Background, and Antibiotic Resistance of *Escherichia coli* Isolated from Turkeys with Airsacculitis

**DOI:** 10.1155/2014/289024

**Published:** 2014-07-02

**Authors:** Marcos Paulo Vieira Cunha, Maria Gabriela Xavier de Oliveira, Mirela Caroline Vilela de Oliveira, Ketrin Cristina da Silva, Cleise Ribeiro Gomes, Andrea Micke Moreno, Terezinha Knöbl

**Affiliations:** ^1^Departamento de Patologia, Faculdade de Medicina Veterinária e Zootecnia, Universidade de São Paulo, Avenida Professor Orlando Marques de Paiva, No. 87, Cidade Universitária, 05508-270 São Paulo, SP, Brazil; ^2^Departamento de Medicina Veterinária Preventiva e Saúde Animal, Faculdade de Medicina Veterinária e Zootecnia, Universidade de São Paulo, Avenida Professor Orlando Marques de Paiva, No. 87, Cidade Universitária, 05508-270 São Paulo, SP, Brazil

## Abstract

Avian Pathogenic* Escherichia coli* (APEC) has been studied for decades because of its economic impact on the poultry industry. Recently, the zoonotic potential of APEC and multidrug-resistant strains have emerged. The aim of this study was to characterize 225 APEC isolated from turkeys presenting airsacculitis. The results showed that 92% of strains presented a multidrug-resistance (MDR), and the highest levels of resistance were to sulfamethazine (94%) and tetracycline (83%). Half of these strains were classified in phylogenetic group B2, followed by B1 (28.6%), A (17.1%), and D (4.8%). The prevalence of virulence genes was as follows: salmochelin (*iroN,* 95%), increased serum survival (*iss,* 93%), colicin V (*cvi/cva,* 67%), aerobactin (*iucD,* 67%), temperature-sensitive haemagglutinin (*tsh,* 56%), iron-repressible protein (*irp2,* 51%), invasion brain endothelium (*ibeA,* 31%), vacuolating autotransporter toxin (*vat,* 24%), K1 antigen (*neuS, *19%), enteroaggregative heat-stable cytotoxin (*astA,* 17%), and pilus associated with pyelonephritis (*papC, *15%). These results demonstrate that the majority of the investigated strains belonged to group B2 and were MDR. These data suggest that turkeys may serve as a reservoir of pathogenic and multidrug-resistance strains, reinforcing the idea that poultry plays a role in the epidemiological chain of ExPEC.

## 1. Introduction 

Extraintestinal pathogenic* Escherichia coli* (ExPEC) infections in poultry production generate a negative economic impact, especially in countries such as Brazil, China, and the United States, where the poultry industry is highly developed [1, 2]. The subpathotype of ExPEC that infects poultry, causing colibacillosis, is known as avian pathogenic* Escherichia coli* (APEC) and is considered a heterogeneous group of pathogens with a broad range of virulence characteristics.

APEC is present in all stages of the poultry production chain, causing several types of lesions and diseases. The most common manifestations associated with the clinical presentation of colibacillosis are airsacculitis, perihepatitis, pericarditis, salpingitis, omphalitis, coligranuloma, cellulitis, swollen head syndrome, and sepsis [2, 3]. In turkeys, especially in young birds, APEC is related to a condition called turkey osteomyelitis complex (TOC), which causes various types of lesions including arthritis/synovitis, soft-tissue abscesses, green discoloration of the liver, and osteomyelitis of the proximal tibia [3, 4]. All of these clinical conditions caused by APEC result in partial or complete condemnation of a large number of carcasses, causing annual losses of million dollars. In Brazil, it is estimated that 4% of the slaughtered poultry are condemned for airsacculitis and 1.3% for lesions caused by systemic colibacillosis [5].

Colonization of the trachea and air sacs is considered the first step of a systemic infection by APEC, followed by the colonization of the liver and the pericardium, with subsequent bacteremia. Airsacculitis is one of the first signs observed in experimental infections [3]. To adhere, colonize, invade, and cause infection, APEC strains employ a diverse set of virulence traits, including adhesins, protectins, iron uptake or transport systems, capsules, elements involved in evasion of the immune response, toxins, and invasins [6–9]. As in human ExPEC, APEC lineages can express a diverse and heterogeneous repertoire of virulence genes [1]. Some studies have associated several virulence genes with the APEC pathotype, and these genes may be present on plasmids or chromosomal DNA fragments called PAIs (pathogenicity islands) [6–10].

Recently, several studies have shown that some APEC clones are very similar to extraintestinal pathotypes that affect humans (UPEC: uropathogenic* Escherichia coli*; NMEC: neonatal meningitis-causing* Escherichia coli*). These APEC strains are indistinguishable from human ExPEC by the possession of certain virulence factors and phylogenetic groups and are able to cause disease in mammalian models of human disease [7, 11–17]. For this reason, some authors pointed to poultry as a reservoir for human ExPEC, suggesting a risk to public health [11–13].

In addition to the economic losses and the risk of transmission to humans,* E. coli* strains isolated from livestock, such as APEC, have a high rate of antimicrobial resistance [1, 18]. Of note, alarming levels of contamination by multidrug-resistant (MDR)* E. coli* strains have been reported in poultry meat [1, 18]. Therefore, the aim of this study was to characterize* E. coli* strains isolated from turkeys condemned for airsacculitis in slaughterhouses, including their virulence genotype, their phylogenetic groups, and their antimicrobial resistance.

## 2. Materials and Methods

### 2.1. Bacterial Strains

A total of 225 nonduplicate strains of* Escherichia coli* were taken from the air sacs of turkeys condemned in abattoirs by airsacculitis. These turkeys were located at eight poultry farms in Brazil. Each sample was cultured on brain heart infusion broth for 18 hours, then plated on MacConkey Agar, and incubated aerobically at 37°C for 24 hours. The colonies that were confirmed by standard biochemical tests were stored at −80°C in Luria Bertani broth with 30% glycerol.

### 2.2. Virulence Genotyping

Polymerase chain reaction (PCR) was used to amplify genes of interest after DNA extraction, according to the protocol described by Boom et al. (1990) [19]. The reaction mixtures (50 *μ*L) contained PCR buffer (1X), MgCl2 (1.5 Mm), 200 mM of each deoxyribonucleotide (dATP, dCTP, dGTP, and dTTP), 50 pmol of each oligonucleotide, 1.0 U of Taq DNA polymerase, autoclaved ultrapure water, and 5 *μ*L of the DNA template. Primer pairs for specific amplification of the* iroN *(salmochelin siderophore receptor)*, iss *(increased serum survival) [20];* papC *(pilus associated with pyelonephritis),* iucD *(aerobactin siderophore synthesis) [10];* tsh *(temperature-sensitive haemagglutinin),* vat *(vacuolating autotransporter toxin) [6];* cvi/cva *(structural genes of colicin V operon),* ibeA *(invasion of brain endothelium) [7];* irp2 *(Iron-repressible protein (yersiniabactin synthesis)) [21];* neuS *(K1 capsular antigen) [22];and* astA *(heat-stable cytotoxin associated with enteroaggregative* E. coli, *EAST 1) [23]are described in the references. The amplification steps were 95°C for 5 min, followed by 30 cycles of 95°C for 60 s, 52–59°C for 60 s (variable for each primer pair), and 72°C for 60 s, with a final cycle of 72°C for 10 min. The amplified products were separated by electrophoresis on a 1,5% agarose gel and stained with SyBR Safe DNA gel stain (Invitrogen, São Paulo, Brazil). A 100 bp DNA ladder was used as a molecular size marker.

### 2.3. Phylogenetic Analysis

The strains were assigned to phylogenetic groups according to the method of Clermont et al. (2000) [24]. This method designates strains to one of four phylogenetic groups (A, B1, B2, or D) based on the presence of two genes (*chuA* and* yjaA*) and a specific DNA fragment (TSPE4.C2).

### 2.4. Antibiotic Resistance

The antibiotic resistance profiles of 225 isolates were determined using a disc diffusion test, according to the standardized protocol of the M31-A3 document issued by the Clinical and Laboratory Standards Institute (CLSI) [25]. The antimicrobial agents tested included amoxicillin, cefotaxime, cefoxitin, ceftiofur, enrofloxacin, erythromycin, gentamicin, nalidixic acid, norfloxacin, streptomycin, sulfamethazine, tetracycline, and trimethoprim-sulfamethoxazole. The* Escherichia coli* ATCC 25922 reference strain was used as quality control in the antibiotic susceptibility tests.

### 2.5. Statistical Analyses

The significance of the results was established using either Fisher's exact test (two-tailed) or *χ* ^2^with the Yates correction, as appropriate. The level for statistical significance was *P* ≤ 0.05.

## 3. Results

**Figure 1:**
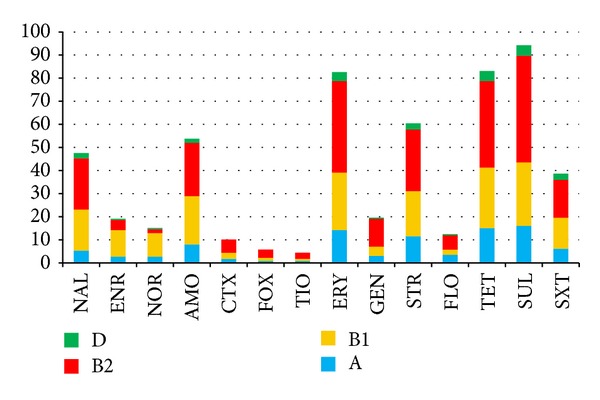
Distribution of 225 APEC strains in relation to antibiotic resistance. NAL: nalidixic acid, ENR: enrofloxacin, NOR: norfloxacin, AMO: amoxicillin, CTX: cefotaxime, FOX: cefoxitin, TIO: ceftiofur, ERY: eritromicin, GEN: gentamycin, STR: streptomycin, FLO: florfenicol, TET: tetracycline, SXT: sulfamethoxazole/trimetropim.

The results showed that 92% of the strains (207/225) presented a multidrug-resistance (MDR) profile (resistant to ≥1 agent in ≥3 antimicrobial categories). The highest rates of resistance were observed with sulfamethazine 94.2% (212/225), tetracycline 83.1% (187/225), and erythromycin 82.6% (186/225). Among the *β*-lactam antibiotics, resistance to amoxicillin was more prevalent (53.7%, 121/225), followed by cefotaxime (10.2%, 23/225), cefoxitin (5.7%, 13/225), and ceftiofur (4.4%, 10/225). Among the quinolones and fluoroquinolones, nalidixic acid resistance was 48% (107/225), followed by enrofloxacin (19%, 43/225) and norfloxacin (15.1%, 34/225). Strains that were resistant to streptomycin and gentamycin comprised 60.4% (136/225) and 19.5% (44/225), respectively (Figure 1). None of strains were sensitive to all antibiotics (ATBs), and the 225 strains presented a resistance average of 5.4 ATBs. The resistance profiles showed a total of 108 different patterns according to the 14 ATB profile combinations, and the pattern of amoxicillin, erythromycin, streptomycin, tetracycline, sulfamethazine, and sulfamethoxazole/trimethoprim resistance was the most prevalent (5.7%, 13/225) (Table 2).

The phylogenetic analysis showed that half of the isolates belonged to the B2 group (112/225), followed by B1 (28.6%, 64/225), A (17.1%, 38/225), and D (4.8%, 11/225) groups. The strains that belonged to phylogroup B2 were associated with more virulence genes, presenting an average of 6.5 virulence genes by strain, while the other groups presented approximately 4.4 genes by strain (Table 1).

The virulence profiles showed that the plasmidial genes* iroN* and* iss* were detected in 214 (95%) and 211 (93%) strains. The other two plasmidial genes,* cvi/cva* and* iucD, *were detected at the same prevalenceof 67% (152/225), followed by genes that encoded the temperature-sensitive haemagglutinin (*tsh*), with a 56% (128/225) prevalence. The* tsh* gene was also positively associated with the B2 group (*P* ≤ 0.05), as well as* vat*,* irp2*,* ibeA,* and* neuS* genes (Table 1). The strains were grouped into 96 distinctive virulence profiles, and the association of nine genes,* vat, irp2, cvi/cva, neuS, iroN, iss, iucD, tsh, *and* ibeA, *was the most prevalent (7.5%). All strains with this virulence profile were included in the B2 phylogroup (Table 2).

Linking antibiotic resistance, virulence profile, and phylogenetic groups, we found that strains susceptible to fluoroquinolones (enrofloxacin and norfloxacin) were positively associated (*P* ≤ 0.05) with virulence genes (*vat, irp2, cvi/cva, neuS, ibeA, *and* tsh*) and the B2 phylogroup, and these strains also presented five or more virulence determinants. On the other hand, the strains resistant to fluoroquinolones were associated with the B1 phylogroup and the strains whose virulence profile presented less than five virulence genes. This association between increased virulence potential and antibiotic susceptibility was also observed in strains susceptible to amoxicillin, streptomycin, tetracycline, and sulfamethoxazole/trimethoprim.

The resistance phenotype associated with increased virulence and the B2 phylogroup were both exhibited by strains resistant to gentamycin (Table 3).

## 4. Discussion 

In the last few decades, many studies have explored the mechanisms behind the pathogenesis and molecular epidemiology of APEC, looking for molecular markers that can contribute to the reduction of losses in poultry production. Recently, studies involving APEC highlight the zoonotic potential of these strains, as many APEC isolates are closely related to human ExPEC (NMEC and UPEC), as well as the problem of multidrug-resistance to antibiotics. To establish virulence profiles, phylogenetic backgrounds, and antibiotic resistance patterns, this study was conducted with strains of* Escherichia coli* isolated from turkeys presenting airsacculitis lesions in slaughterhouses.

The presence of either the ColV plasmid or the sequences associated with this plasmid has been associated with APEC virulence [9, 20]. This study showed a high prevalence of genes encoded by this plasmid as* iroN, iss, iucD, tsh,* and* cvi/cva* (Table 1).* iroN* and* iss* were present in more than 93% of strains. Johnson et al. [9] identified five plasmidial genes, including* iroN *and* iss, *associated with high pathogenicity strains and used this criterion for the differentiation of pathogenic strains and fecal* E. coli.* A study conducted in Korea between 1985 and 2005 showed that the prevalence of* iroN* in APEC was 100% in all periods, whereas the prevalence of the* iss* gene was variable and increasing as the years passed [26].

P-fimbriae (*pap* (pyelonephritis-associated pili)) are an important virulence factor of ExPEC and are common among UPEC strains isolated from humans and dogs [14, 27]. In our study, the prevalence of the* papC* gene (15%) was lower than that in previous studies developed in Germany [6, 7] and in the United States [14, 15] but was very similar when compared with Brazilian prevalence, detected in a study with APEC isolated from poultry [28].

The iron uptake systems are also present in ExPEC, and some strains have developed more than one strategy for sequestering iron from their hosts [29]. In this study, we examined three genes associated with iron uptake. In addition to the* iroN* gene, which was previously discussed, we surveyed* iucD *and* irp2*, which are present in 51% and 67% of strains, respectively. This prevalence is in agreement with epidemiological surveys performed in Asia and Europe [6, 7, 10, 26].

The genes associated with invasion,* ibeA* and* neuC,* are generally present in NMEC strains and play a role in the pathogenesis of neonatal meningitis [7, 15, 30, 31]. Comparative studies involving APEC and NMEC showed that APEC strains harboring these two genes were classified into the B2 phylogroup, revealing an overlap of virulence properties and serogroups and indicating a close relationship with NMEC strains [7, 15, 30]. Germon et al. [31] highlighted the* ibeA* gene's role in the pathogenesis of colibacillosis through invasion assays. After the deletion of the* ibeA* gene, these authors noted a decrease in virulence. In the present study, the strains positive for the* ibeA* and* neuC* genes had a prevalence of 31% and 19%, respectively, and were associated with the B2 phylogroup (*P* ≤ 0.05) (Table 1). They were also correlated with strains that presented a high number of virulence factors (Table 2).

Epidemiological surveys in many countries, including Brazil, have classified most APEC strains into phylogroups A and D [7, 9, 15, 32]. Human ExPEC belonged almost exclusively to B2 and less so to the D group [24, 27]. Our results showed that most of the strains isolated from turkeys with airsacculitis belonged to group B2 (50%), followed by B1 (28.5%), A (17%), and D (4.5%). Phylogroup B2 is considered more virulent in ExPEC infection and frequently had the greatest number of virulence genes [1, 27]. Strains belonging to the B2 group in this study presented the highest average of genes by strain (6.5), when compared with the virulence scores from other groups (Table 1).

The B2 strains are also associated with the fluoroquinolone, amoxicillin, tetracycline, and sulfamethoxazole/trimethoprim susceptible pattern, while the strains resistant to these antibiotics were associated with the B1 phylogroup (Table 3). Other authors have reported that the ExPEC strains belonging to the B2 phylogroup are susceptible to antibiotics, especially quinolones [33, 34].

Some authors have discussed the relationship between low virulence and resistance to quinolones and fluoroquinolones in human ExPEC. Several hypothetical mechanisms have been posited to explain this characteristic as the loss or deletion of pathogenicity island (PAIs) and plasmids after drug exposure: an incompatibility of conjugative plasmids, a loss of resistance plasmids after the acquisition of virulence plasmids, and a predisposition, among lower virulence strains and non-B2-strains, for acquiring resistance [35–37]. However, Johnson et al. [33, 38] assigned this fact to the importation of strains from an animal reservoir, due to selective antibiotic pressure associated with antibiotic use as growth promoters, which selects for resistant strains but with features of low virulence.

As the association between high virulence and fluoroquinolone susceptibility, the tetracycline-susceptible strains were related to the B2 phylogroup and presented the* vat, irp2, cvi/cva, neuS,* and* ibeA* genes (Table 3). Starčič Erjavec et al. [37] showed that tetracycline-resistant UPEC was less virulent, while susceptible strains presented higher virulence potential and belonged to the B2 group, corroborating our findings in APEC isolated from turkeys.

On the other hand, the strains that presented a gentamicin resistance phenotype in our study were positively associated with group B2 and multiple virulence genes (*vat*,* irp2, cvi/cva, neuS, iucD, *and* tsh*) (Table 3). Altekruse et al. [39] compared* E. coli* isolated from turkeys with colibacillosis and fecal strains isolated from healthy turkeys and found the highest level of gentamycin resistance in sick birds. Lay et al. [40] showed the same association between virulence factors in streptomycin- and gentamycin-resistant strains isolated from fecal samples of healthy pigs. These authors attribute this coexistence of virulence genes and resistance to aminoglycosides to the same genetic elements, such as plasmids or transposons [40]. Comparing our results with the results of these authors, we suggested that the selective pressure caused by antibiotic use in poultry production could be selecting virulent strains that possess aminoglycoside genes.

The emergence and selection of resistant bacteria are considered by many authors to be a direct consequence of antibiotic use in animal production. Some studies demonstrate that the introduction of an antibiotic in veterinary practice is correlated with the growth of resistant bacteria in an animal's fecal microbiota. Theoretically, these agents could be transmitted from humans through the food chain and spread to human communities and hospitals [41]. Intensive systems of poultry and pig farming in many countries are still dependent on the use of subtherapeutic antibiotics, and this practice has been questioned because of the danger of selecting multidrug-resistance bacteria and consequently impacting public health [1]. In this study, the level of multidrug-resistance (resistant to ≥1 agent in ≥3 antibiotic categories) was 92% (207/225). This proportion was also described by Zhao et al. [42] in a study of 95 APEC strains in the United States. The highest resistance levels described by Zhao et al. [42] were to sulfamethazine and tetracyclines, as in our study (Figure 1). Comparing our results with a study of APEC in China, it is possible to observe that they also have high levels of resistance to tetracycline, as opposed to the enrofloxacin resistance rates of 90% in China and only 19% in this study [43]. A survey in Australia found resistance to sulfas, tetracycline, streptomycin, and florfenicol at lower levels than in our results [44]. This fact may be related to restrictions about the use of drugs in Australia, with stricter legislation in the poultry chain.

In Brazil eleven antimicrobials are authorized as growth promoters in turkey feed (virginiamycin, avilamycin, enramycin, bacitracin methylene disalicylate, zinc bacitracin, colistin, chlorhexidine, lincomycin, flavomycin, halquinol, and tylosin) [45]. Antimicrobials such as colistin, lincomycin, and bacitracin are largely used in human medicine, and strains resistant to these antibiotics may be being selected in Brazilian poultry industry. This fact reaffirms the importance of responsible use of antimicrobials in poultry production. Lima-Filho et al. [46] assessed the zoonotic potential of multidrug-resistant ExPEC strains isolated from healthy poultry carcass in Brazil. These authors reported that a strain resistant to 11 antibiotics and harboring* iss* gene was able to cause a severe degeneration of hepatocytes and spleen when inoculated in mice. However, an* E. coli* strain resistant to two antibiotics that presented the same virulence profile did not cause injury in mice. Linking our results with these observations highlights multidrug-resistant ExPEC strains more than 92%, indicating public health concern in meat and poultry production in Brazil.

## 5. Conclusion

Data from this study revealed a high prevalence of B2 phylogroup strains that also possess virulence genes, such as* ibeA* and* neuS*. The similarity of these strains to human ExPEC makes these data relevant and alerts to the possibility of these birds acting as reservoirs of bacteria that pose a danger to public health.

Strains more virulent were correlated with a susceptible antimicrobial phenotype, in contrast to isolates that presented resistance to enrofloxacin, norfloxacin, amoxicillin, streptomycin, tetracycline, and sulfamethoxazole/trimetropim which was associated with low virulence profile. Concern about the prevalence of antibiotic resistance is growing worldwide and could be associated with indiscriminate use of these medications in animal production. The rational and restricted use in turkey production is needed, as is the implementation of strategies for monitoring and surveillance of these strains that are potentially pathogenic for humans.

## Figures and Tables

**Table 1:** Distribution of 225 *E*. *coli* strains according to the phylogenetic group and presence of virulence genes.

Group (*n*)	Prevalence *n* (%)											
	*ir* *oN*	*is* *s*	*cv* *i*/*cva*	*ts* *h*	*va* *t*	*iu* *cD*	*ir* *p*2	*ne* *uS*	*ib* *eA*	*pa* *pC*	*as* *tA*	*VS* ^#^
A (38)	33 (87)	37 (97)	27 (71)	19 (48)	1 (2)	24 (63)	15 (38)	0 (0)	3 (8)	1 (2)	5 (13)	4.3
B1 (64)	62 (96)	59 (92)	38 (59)	24 (38)	1 (1)	43 (67)	14 (22)	2 (3)	16 (25)	8 (12)	8 (14)	4.2
B2 (112)	109 (97)	105 (93)	81 (72)	80 (71)∗	52 (46)∗	79 (70)	85 (75)∗	40 (36)∗	50 (45)∗	24 (21)	24 (21)	6.5
D (11)	10 (90)	10 (90)	6 (54)	5 (45)	2 (18)	6 (54)	3 (27)	1 (9)	3 (27)	1 (9)	2 (18)	4.4

Total (225)	214 (95)	211 (93)	152 (67)	128 (56)	56 (24)	152 (67)	116 (51)	43 (19)	71 (31)	34 (15)	39 (17)	5.4

*Positive association between virulence factor and phylogenetic group (*P* ≤ 0.05).

^#^VS: virulence score. A virulence score was calculated for each isolate as the sum of all virulence factors.

*P* value was determined using Fisher's exact test for comparisons among virulence factors and phylogenetic groups.

**Table 2:** Virulence and antibiotic resistance profiles in 225 strains of *E*. *coli*.

Virulence profiles	Phylogroup (*n*)	Total *n* (%)
*vat, irp2, cvi/cva, neuS, iroN, iss, iucD, tsh, ibeA *	B2 (17)	17 (7.5)
*irp2, cvi/cva, iroN, iss, iucD, tsh *	A (12), B1 (2), B2 (2)	16 (7.1)
*cvi/cva, iroN, iss *	A (5), B1 (5), B2 (3), D (1)	14 (6.2)
*irp2, cvi/cva, neuS, iroN, iss, iucD, tsh, ibeA *	B2 (8)	8 (3.5)
*iroN, iss *	A (3), B1 (4), B2 (1)	8 (3.5)
*cvi/cva, iroN, iss, iucD, tsh *	A (2), B1 (4), B2 (1)	7 (3.1)
*cvi/cva, iroN, iss, iucD *	B1 (6)	6 (2.6)
*iroN, iss, iucD *	B1 (3), B2 (1), D (1)	5 (2.2)
*irp2, iroN, iss, iucD *	B1 (4), B2 (1)	5 (2.2)
*vat, irp2, cvi/cva, iroN, iss, iucD, tsh, ibeA *	B2 (4), D (1)	5 (2.2
Other 86 combinations	A (16), B1 (36), B2 (74), D (8)	134 (59.5)

≥4 genes	A (24), B1 (45), B2 (99), D (8)	176 (78.3)
≥6 genes	A (14), B1 (13), B2 (79), D (2)	108 (48)
≥8 genes	B2 (43), D (1)	44 (19.6)

ATB resistance profiles	Phylogroup (*n*)	Total *n* (%)

AMO, ERY, STR, TET, SUL, SXT	A (6), B1 (3), B2 (4)	13 (5.7)
NAL, ENR, NOR, AMO, ERY, STR, TET, SUL	A (3), B1 (8), D (1)	12 (5.3)
ERY, TET, SUL	A (4), B1 (3), B2 (2), D (1)	10 (4.4)
NAL, AMO, ERY, STR, TET, SUL, SXT	A (1), B1 (4), B2 (4)	9 (4)
ERY, SUL	B1 (1), B2 (6)	7 (3.1)
NAL, ENR, NOR, AMO, ERY, STR, TET, SUL, SXT	B1 (6)	6 (2.7)
AMO, ERY, STR, TET, SUL	A (1), B1 (1), B2 (4)	6 (2.7)
AMO, STR, TET, SUL, SXT	B1 (2), B2 (3), D (1)	6 (2.7)
ERY, STR, TET, SUL	B1 (1), B2 (4), D (1)	6 (2.7)
ERY, STR, TET, SUL, SXT	A (2), B1 (1), B2 (1), D (1)	5 (2.2)
Other 98 combinations	A (21), B1 (34), B2 (84), D (6)	145 (64.5)

≥3 ATB	A (38), B1 (62), B2 (98), D (11)	209 (92.9)
≥5 ATB	A (24), B1 (53), B2 (35), D (5)	117 (52)
≥8 ATB	A (7), B1 (20), B2 (13), D (1)	41 (18.3)

**Table 3:** Distribution of virulence genes and phylogenetic groups in relation to antibiotic resistance phenotypes among 225 ExPEC isolates.

Antibiotic	NAL^a^		ENR		NOR		AMO		CTX		FOX		TIO		ERY		GEN		STR		FLO		TET		SXT	
Virulence factor	*R* ^b^	*S*	*R*	*S*	*R*	*S*	*R*	*S*	*R*	*S*	*R*	*S*	*R*	*S*	*R*	*S*	*R*	*S*	*R*	*S*	*R*	*S*	*R*	*S*	*R*	*S*
*astA *																			++							
*vat *				+		+		++									+			++				++		+
*irp2 *				+		++		++									+			+				+		+
*cvi/cva *	+^c^			+		+		+					+				+			+	+			+		+
*neuS *				+		+			+		+		+				+							+		
*iroN *																										
*iss *						+																				
*papC *																			+							
*ibeA *				+		+																		+		
*iucD *																	+									
*tsh *				+		+		+								+	+									+

MV^d^ (≥5 VF)				+		+		+									+									+
Non-MV (<5)			+		+		+											+							+	

Phylogroup																										
A		+																								
B1	+		++		++		+																+			
B2				++		++		+									+							+		
D																										

Virulence score	5.5	5.2	4.3	5.6	4.1	5.6	4.9	5.9	5.4	5.4	5.7	5.3	6.6	5.3	5.3	5.8	6.7	5	5.2	5.6	6.1	5.3	5.1	6.5	4.8	5.7

^a^NAL: nalidixic acid, ENR: enrofloxacin, NOR: norfloxacin, AMO: amoxicillin, CTX: cefotaxime, FOX: cefoxitin, TIO: ceftiofur, ERY: erythromycin, GEN: gentamycin, STR: streptomycin, FLO: florfenicol, TET: tetracycline, SXT: sulfamethoxazole/trimetropim. Sulfamethazine is not shown in the table because it was not associated with any trait of virulence.

^b^ *R*: resistant, *S*: susceptible.

^c^Positive association between traits (“+” = *P* < 0.05; “++” = *P* < 0.0001). Only statistically significant differences are shown. ^d^MV: multivirulent.
